# Performance and exhaust emission characteristics of variable compression ratio diesel engine fuelled with esters of crude rice bran oil

**DOI:** 10.1186/s40064-016-1945-7

**Published:** 2016-03-08

**Authors:** Mohit Vasudeva, Sumeet Sharma, S. K. Mohapatra, Krishnendu Kundu

**Affiliations:** Mechanical Engineering Department, Baddi University of Emerging Sciences and Technology, Baddi, India; Department of Mechanical Engineering, Thapar University, Patiala, India; Biofuel Department, Mechanical Engineering Research and Development Organization, Ludhiana, India

**Keywords:** Crude rice bran biodiesel, Variable compression ratio, Engine performance, Cylinder pressure, Exhaust emissions

## Abstract

As a substitute to petroleum-derived diesel, biodiesel has high potential as a renewable and environment friendly energy source. For petroleum importing countries the choice of feedstock for biodiesel production within the geographical region is a major influential factor. Crude rice bran oil is found to be good and viable feedstock for biodiesel production. A two step esterification is carried out for higher free fatty acid crude rice bran oil. Blends of 10, 20 and 40 % by vol. crude rice bran biodiesel are tested in a variable compression ratio diesel engine at compression ratio 15, 16, 17 and 18. Engine performance and exhaust emission parameters are examined. Cylinder pressure-crank angle variation is also plotted. The increase in compression ratio from 15 to 18 resulted in 18.6 % decrease in brake specific fuel consumption and 14.66 % increase in brake thermal efficiency on an average. Cylinder pressure increases by 15 % when compression ratio is increased. Carbon monoxide emission decreased by 22.27 %, hydrocarbon decreased by 38.4 %, carbon dioxide increased by 17.43 % and oxides of nitrogen as NO_x_ emission increased by 22.76 % on an average when compression ratio is increased from 15 to 18. The blends of crude rice bran biodiesel show better results than diesel with increase in compression ratio.

## Background

Fossil fuels are the major source of energy worldwide. The cheaper cost of diesel as an automotive fuel compared to gasoline has received increased attention, which further has resulted in the increase of on road mid-size diesel vehicles (Sedan and Hatchback class). This increase in number of vehicles on road along with uncontrolled emissions from motor vehicles has majorly affected the concentration of air pollution, raising serious environmental issues.

The leading cause of death worldwide is air pollution according to World Health Organisation (WHO). The 2014 version of the Ambient Air Pollution (AAP) (AAP 2014) in cities database released by WHO ranks New Delhi (National capital of India) as the most polluted city in the world. New Delhi has the worst air conditions in the world measuring the concentration of air pollution at 153 micrograms per cubic meter (µg/m^3^) of air, which is far much greater than normally considered safe air (31–60 µg/m^3^). Also half of the top 20 most polluted cities in world are in India only. With the continuous increase in energy demands, concentration of air pollution and limited availability of fossil fuels, the need for alternative environmental friendly renewable sources of energy have come into focus. Being biodegradable, non-toxic and environmental friendly in nature, biodiesel has received increased attention as an alternative renewable source. Oils and fats are the major sources of biodiesel production (Balat and Balat 2008; Schuchardta et al. 1998). Vegetable oils can be introduced in engine directly, however due to high viscosity it results in poor atomization, injection and combustion problems. In order to reduce viscosity of the vegetable oils, trans-esterification is carried out (Fukuda et al. 2001; Ma and Hanna 1999; Gerpen 2005; Leung et al. 2010).

With India being a major petroleum importing country, the choice of feedstock for production of biodiesel within the geographical region is an economical viable option, as approximately 60–70 % of biodiesel cost is attributed to raw material (Demirbas 2007; Phan and Phan 2008). Higher Free Fatty Acid (FFA) crude rice bran oil is one of the potential sources for production of biodiesel (Balat 2011; Ju and Vali 2005). It is extracted from rice bran which is a byproduct of rice processing industry (Kusum et al. 2011). Crude rice bran oil having lower FFA is used for production of edible grade rice bran oil. India ranks among the leading rice producing countries in the world and is capable of producing around 6 million tonnes of oil. Current production capacity is limited to 0.4 million tonnes, half of it being from edible grade and rest being with higher FFA which is left unutilized (Kusum et al. 2011). For higher FFA content oils esterfication is carried out in two stages. Firstly an acid catalyzed esterification followed by an alkali catalyzed trans-esterification is adopted (Gerpen 2005; Leung et al. 2010; Canakci and Gerpen 2001; Ramadhas et al. 2005). Thus as a low cost feedstock, the unutilized rice bran oil can be used for biodiesel production and used as an alternative cheap and environmental friendly fuel in India.

Use of alternative environment friendly fuels along with improvements in the engine design can result in better engine performance and lower exhaust emissions. Automobile vehicles with diesel engine usually are operating with compression ratio in the range of 15–18. Experimental work has been done (Sinha and Agarwal 2007; Lin et al. 2009; Saravanam et al. 2010) to study the engine characteristics of a diesel engine at a single compression ratio fuelled with blends of rice bran oil biodiesel. Thus, realizing the importance and potential of rice-bran oil in catering to the energy needs and environmental issues of the country, an effort has been made in the present work to investigate the variation in engine performance and exhaust emission characteristics of a 4-stroke diesel engine fuelled with blends of crude rice bran biodiesel by varying compression ratio from 15 to 18.

## Methods

**Fig. 1:**
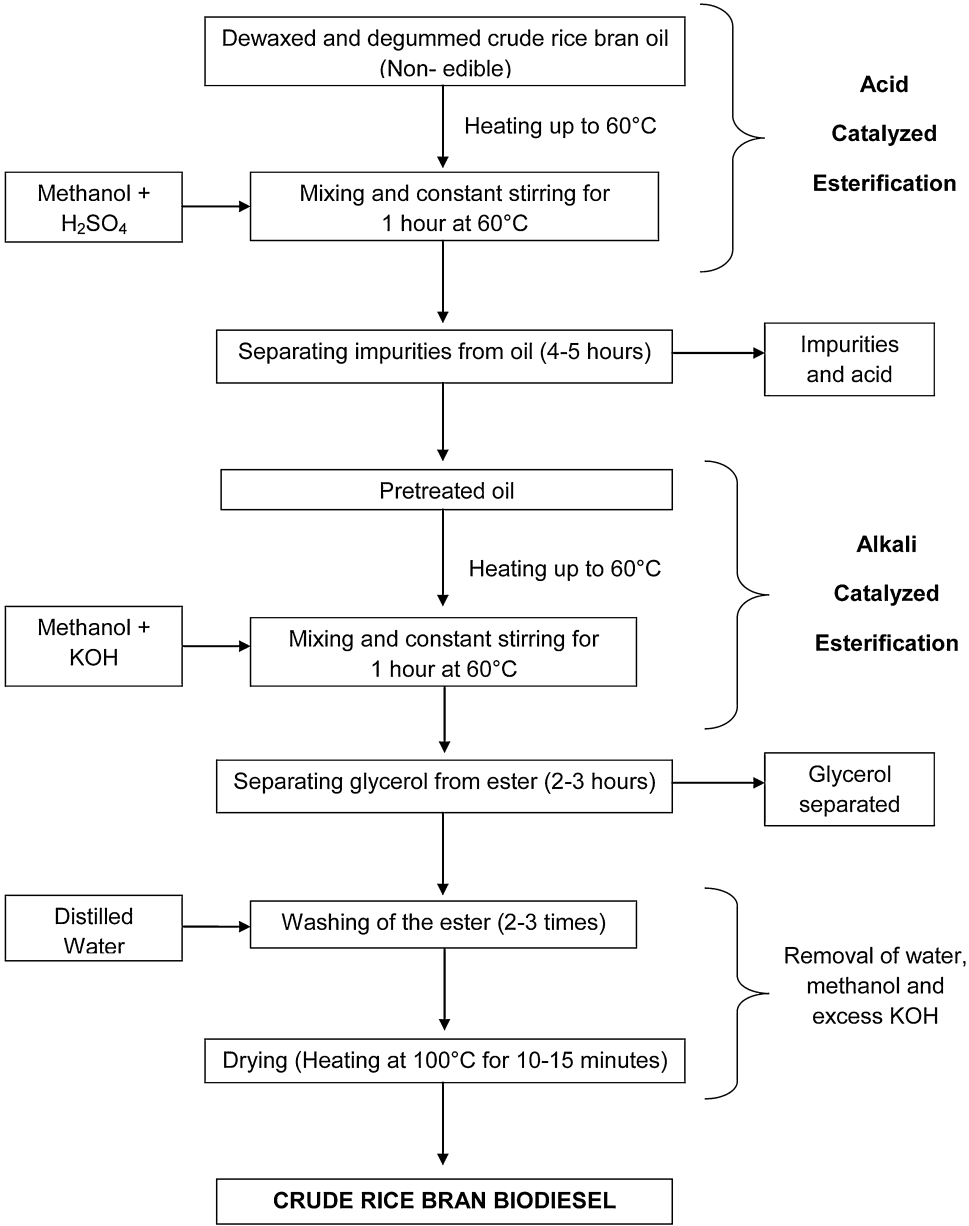
Trans-esterification process for crude rice bran biodiesel production

Methanol, potassium hydroxide (KOH) and sulphuric acid (H_2_SO_4_) were used for carrying out a 2-step esterification in water bath shaker. An acid catalyzed esterification followed by an alkali catalyzed esterification was carried out for higher FFA crude rice bran oil. Figure 1 shows the transesterification process carried out for biodiesel production from crude rice bran oil. Table 1 shows the physical and chemical properties of crude rice bran oil and Table 2 shows the various properties of the prepared biodiesel. Cetane index of diesel and biodiesel blends is calculated by a four variable equation as per ASTM D4737-10 (2010) standard test method. Cetane index calculated for diesel, 10, 20 and 40 % vol. crude rice bran biodiesel blends are 49.5, 51.4, 52.1 and 54 respectively.

**Table 1:** Physical and chemical properties of crude rice bran oil

*Properties*	
C14:0 Myristic acid (saturated), %	0.34
C16:0 Palmitic acid (saturated), %	19.5
C18:0 Stearic acid (saturated), %	2.3
C18: 1 Oleic acid (monosaturated), %	43
C18: 2 Linolic acid (polysaturated), %	32
C18: 3 Linolenic acid (polysaturated), %	1.6
C20 Arachidic acid (saturated), %	0.7
C22 Higher fatty acids, %	0.6
FFA content, %	16
Density at 15 °C, kg/m^3^	920
Iodine value	96
Saponification value	187
Moisture, %	0.8
Oryzanol, %	2.0

**Table 2:** Crude rice bran biodiesel properties

Property parameters	Crude rice bran biodiesel
Density at 15 °C (kg/m^3^)	877
Viscosity at 40 °C (mm^2^/s)	3.57
Carbon Residue (% w/w)	0.244
Flash point (°C)	210
Calorific value (MJ/kg)	41.08
FFA content (%)	0.25
Cloud point (°C)	0
Pour point (°C)	−4
Free glycerol (% w/w)	0.02
Glycerol (% w/w)	0.21

A 4-stroke single cylinder variable compression ratio (VCR) compression ignition direct injection (DI) engine is used for the test. The experimental set-up has the instrumentation (Piezo sensor) for measuring the cylinder pressure variation with crank angle for every 1° increment. Lab-view^®^ based software “Enginesoft” (http://www.apexinnovations.co.in/) is used for acquiring the data which acts as interface between engine and the user. The compatible multifunction data acquisition module for USB used is “NI USB-6210” (http://sine.ni.com/nips/cds/view/p/lang/en/nid/203223). Each engine test data obtained is conditioned and processed for 10 cycles, which means that at a particular engine load condition “Enginesoft” gives each reading after processing it for 10 cycles. An eddy current dynamometer is used for measuring engine torque. Cooling water flow rate was kept constant at 300 L/h (8.33 × 10^−5^ m^3^/s). Table 3 shows the detailed specification of the test set-up. Horiba analyzer (http://www.horiba.com/in/) is used for determination of the unburnt hydro-carbons (HC) in the exhaust. In addition, KM9106 Quintox flue gas analyzer (http://www.kane.co.uk/online-catalogue/emissions-monitoring/km9106) is used for NO_x_, CO_2_ and CO emissions in the exhaust. Sensors of the exhaust gas analyzers are installed in the exhaust pipe at the outlet.

**Table 3:** Specification of test set-up

Make type	Kirloskar
Engine type	Single cylinder 4-stroke DI, water cooled
Compression ratio	Ranging from 12 to 18
Rated power	3.75 kW@1500 R.P.M
Stroke	110 mm
Bore	87.5 mm
Connecting rod length	234 mm
Injection nozzle	3 hole
Fuel injection pressure	195 bar
Injection timing	23° bTDC
Loading device	Eddy current dynamometer(water cooled)
Piezo sensor	Range 5000 PSI
Crank angle sensor	Resolution 1 Deg, Speed 5500 R.P.M with TDC pulse
Data acquisition device	NI USB-6210, 16-bit, 250 kS/s
Load sensor	Load cell, type strain gauge, range 0–50 kg
Fuel flow transmitter	DP transmitter, range 0–500 mm WC
Air flow transmitter	Pressure transmitter, range 0–250 mm WC
Software	“Engine softLV” Engine performance analysis software

To prove the accuracy of the experiment an uncertainty analysis is necessary, as uncertainties and errors may occur due to instrument selection, calibration, working condition, observation and method of conducting the test (Panwar et al. 2010). Percentage uncertainty (±4.33 %) of the engine test data (cylinder pressure-crank angle variation @ 1500 r.p.m) obtained after conditioning and processing 10 cycles is calculated using roots sum square method (Doebelin and Manik 2007). Table 4 shows the gas specifications of the exhaust gas analyzers mentioning the accuracy of the measured parameters.

**Table 4:** Gas specifications

Gas	Range	Resolution	Accuracy
CO	0–10,000 ppm	1 ppm	20 ppm < 400 ppm ±5 % < 2000 ppm ±10 % > 2000 ppm
CO_2_	10 %	0.01 %	±500 ppm < 1 % ±5 % > 1 %
HC	0–5000 ppm	10 ppm	±1.7 % of reading
NO	5000 ppm	1 ppm	±5 ppm < 100 ppm ±5 % > 100 ppm
NO_2_	1000 ppm	1 ppm	±5 ppm < 100 ppm ±5 % > 100 ppm

## Results and discussion

Engine performance and exhaust emissions test results for blends of 10, 20 and 40 % crude rice bran biodiesel (CB10, CB20, CB40) along with diesel are examined at compression ratio (C.R) 15, 16, 17 and 18.

**Fig. 2:**
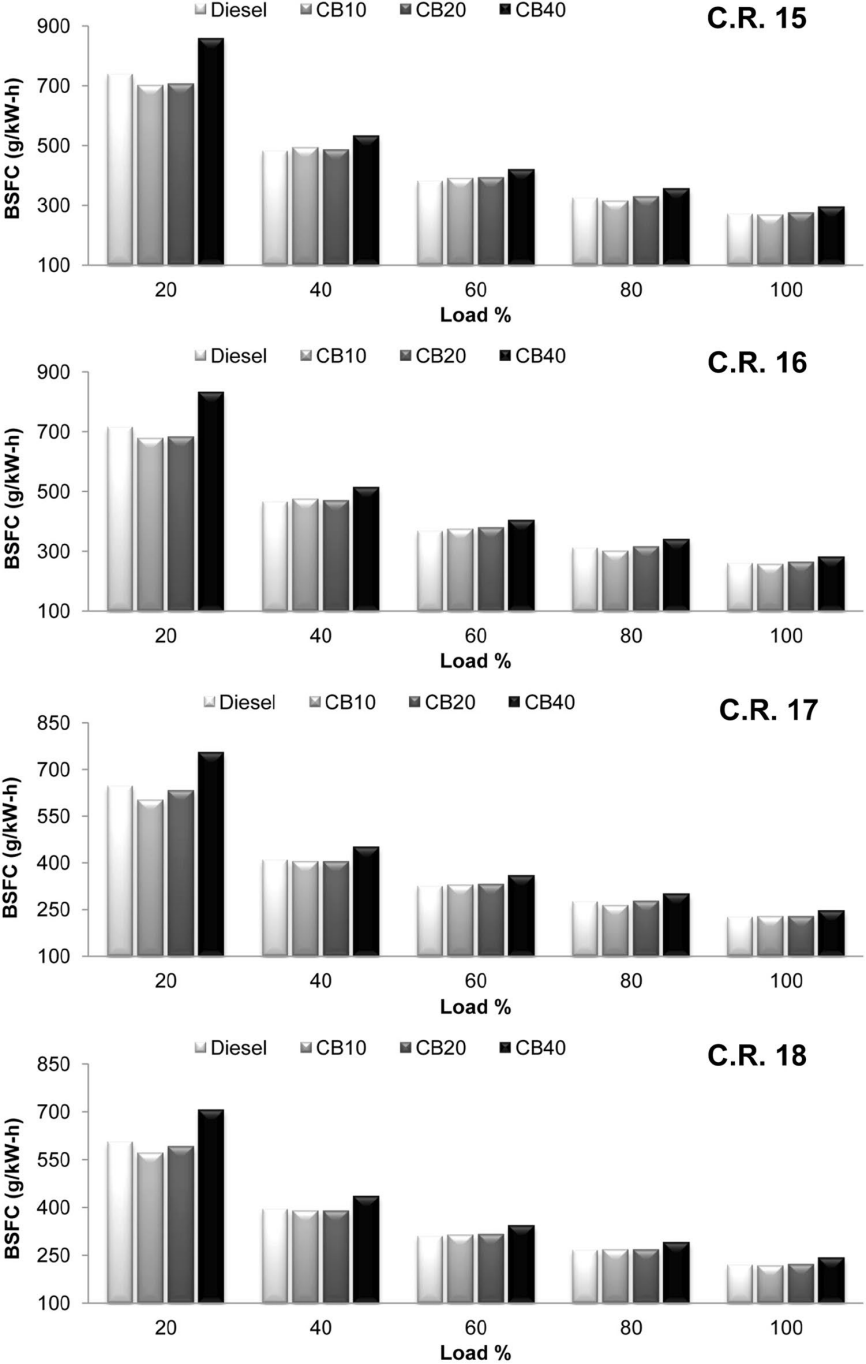
Variation of brake specific fuel consumption with load at compression ratio 15, 16, 17 and 18

Brake specific fuel consumption (B.S.F.C) variation with load at all C.R is shown in Fig. 2. Similar B.S.F.C is observed for CB10, CB20 and diesel. Only CB40 (higher blend ratio) has higher value of B.S.F.C at all C.R. Increase in the amount of crude rice bran biodiesel (lower calorific value and high viscosity fuel) in the blend of diesel and crude rice bran biodiesel reduces the calorific value of the blend and increases its viscosity. Higher fuel viscosity results in poor atomization of the fuel and improper mixing. Thus at a particular engine load condition, an engine running with a lower calorific value and higher viscosity fuel will have increased fuel consumption resulting in higher B.S.F.C, as B.S.F.C is the ratio of fuel consumption to brake power of the engine. For higher blend ratio, lower calorific value and high fuel viscosity can be attributed for higher B.S.F.C. With the increase in compression ratio B.S.F.C decreases for all blends. On an average the decrease in B.S.F.C for diesel, CB10, CB20 and CB40 is 18.42, 18.75, 18.97 and 18.28 % respectively, when C.R is increased from 15 to 18. With the increase in compression ratio, B.S.F.C for biodiesel blends decreases more compared to diesel except for CB40. This improved combustion at higher C.R for biodiesel blends than diesel may be due to their lower volatility.

**Fig. 3:**
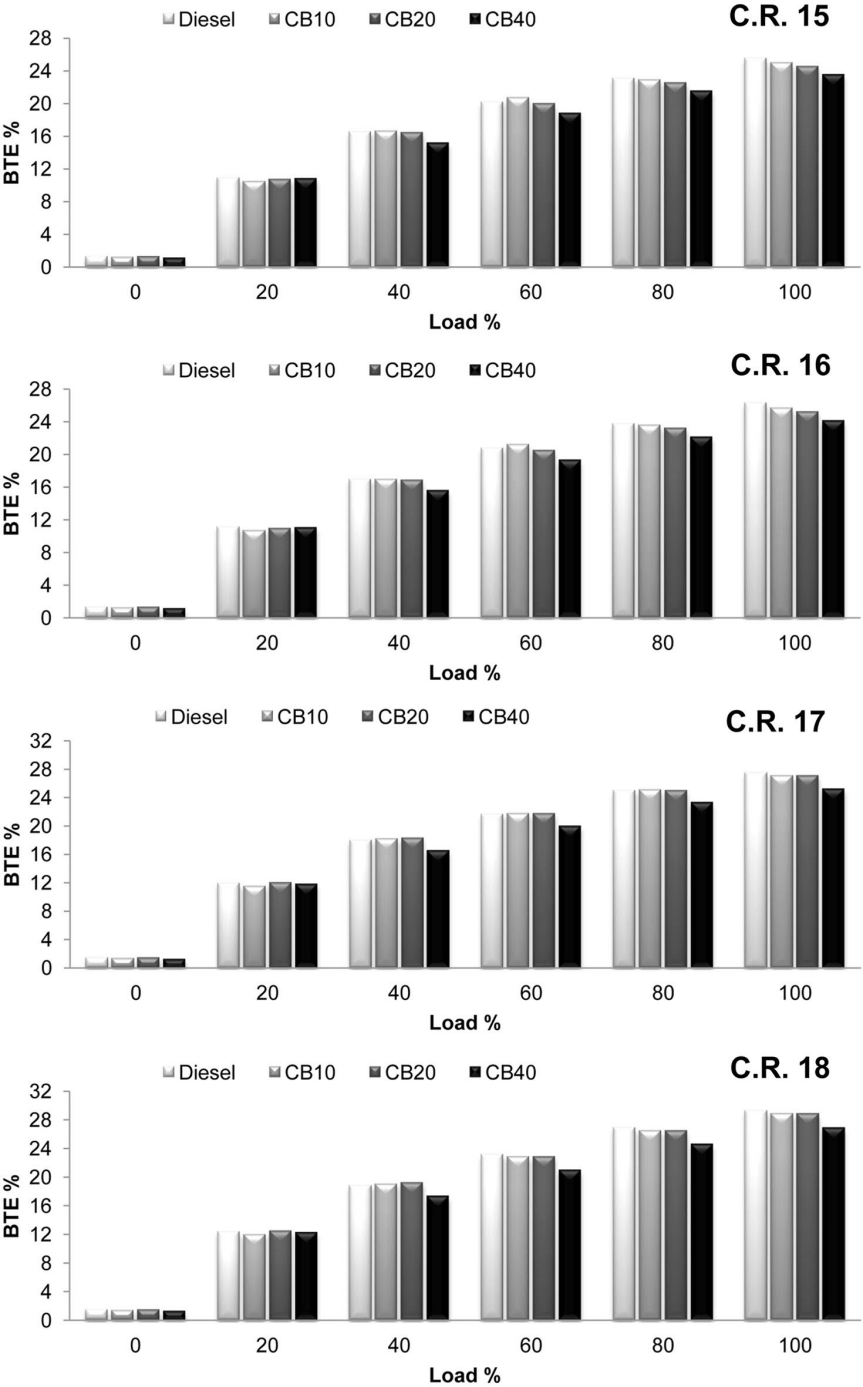
Variation of brake thermal efficiency with load at compression ratio 15, 16, 17 and 18

**Fig. 4:**
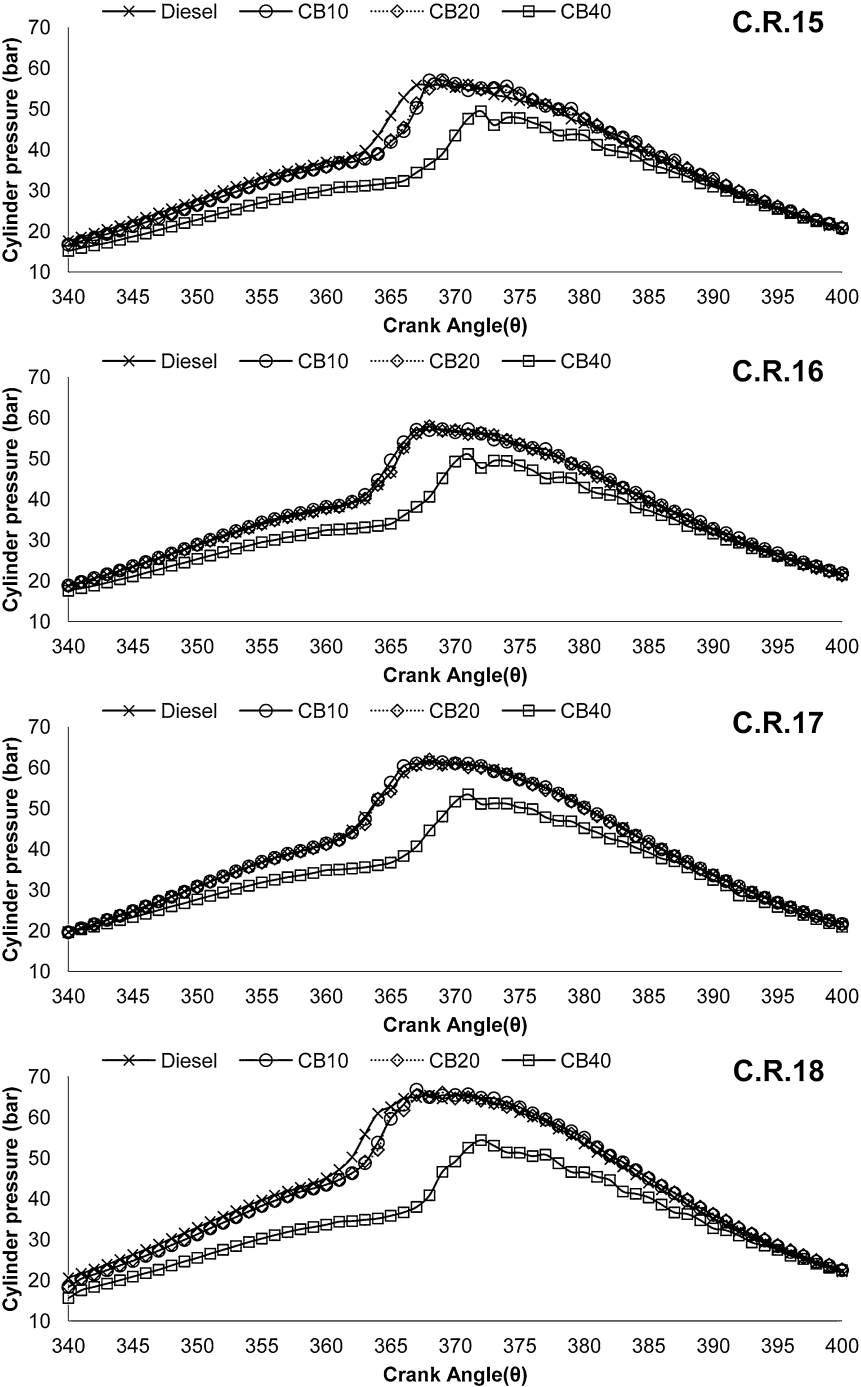
Cylinder pressure variation with crank angle at compression ratio 15, 16, 17 and 18

Brake thermal efficiency (B.T.E) variation with load at all C.R is shown in Fig. 3. Almost similar (slightly lower) values of B.T.E are observed for CB10 and CB20 compared to diesel. Only CB40 has lower B.T.E than others. Higher B.S.F.C is a result of high fuel consumption at a particular engine load condition. As brake thermal efficiency is the ratio of brake power to the product of fuel consumption and calorific value, higher B.S.F.C can be attributed to the reason of lower B.T.E for higher blends of biodiesel. B.T.E increased for all blends with increase in compression ratio. Average increase in B.T.E for diesel, CB10, CB20 and CB40 is 14.43, 14.21, 16.44 and 13.56 % respectively, is recorded when C.R is increased from 15 to 18. Combustion chamber temperature in a diesel engine lies in the range of 1900–2050 °C. With increase in compression ratio, temperature inside the cylinder increases. High combustion temperature due to higher C.R and increased amount of oxygen content in the fuel along with lower volatility of biodiesel blends may be the result of higher increase in B.T.E for biodiesel blends. Cylinder pressure variation with crank angle (P-θ diagram) under full load@1500 r.p.m at all C.R is shown in Fig. 4. Higher cylinder peak pressure along with shorter ignition delay (in terms of attainment of peak pressure w.r.t crank angle) is observed for CB10 and CB20 than diesel. This can be attributed to the better intermixing of air-fuel mixture during the initial stage of combustion resulting in higher peak pressure near to T.D.C in the expansion stroke. At C.R 15 diesel attained a peak pressure of 55.98 bar @ 11° A.T.D.C, whereas CB10 and CB20 attained a peak pressure of 57 and 56.7 bar respectively @ 8° A.T.D.C. As biodiesel is an oxygenated fuel, the combustion reaction takes place at a greater rate due to the presence of excess oxygen, which in turn reduces the ignition delay resulting in early combustion for CB10 and CB20. Cylinder pressure increased with increase in compression ratio. Increase in cylinder pressure for diesel, CB10, CB20 and CB40 is 16.75, 17.04, 16.44 and 9.98 % respectively, is recorded when C.R. is increased from 15 to 18. At C.R 18 the peak pressure for diesel is attained @ 8° A.T.D.C, whereas for CB10 the peak pressure attained is @ 6° A.T.D.C. This increase can be attributed to the rise in combustion temperature due to increase in compression ratio resulting in better and early combustion. For CB40, peak pressure attained in the expansion stroke is lower and further away from T.D.C (11° A.T.D.C) compared to CB10 and CB20 at all C.R. As discussed earlier, higher fuel viscosity and lower calorific value of the higher biodiesel blends results in slower mixing and poor atomization during the initial stages of combustion. This results in lower cylinder peak pressure.

**Fig. 5:**
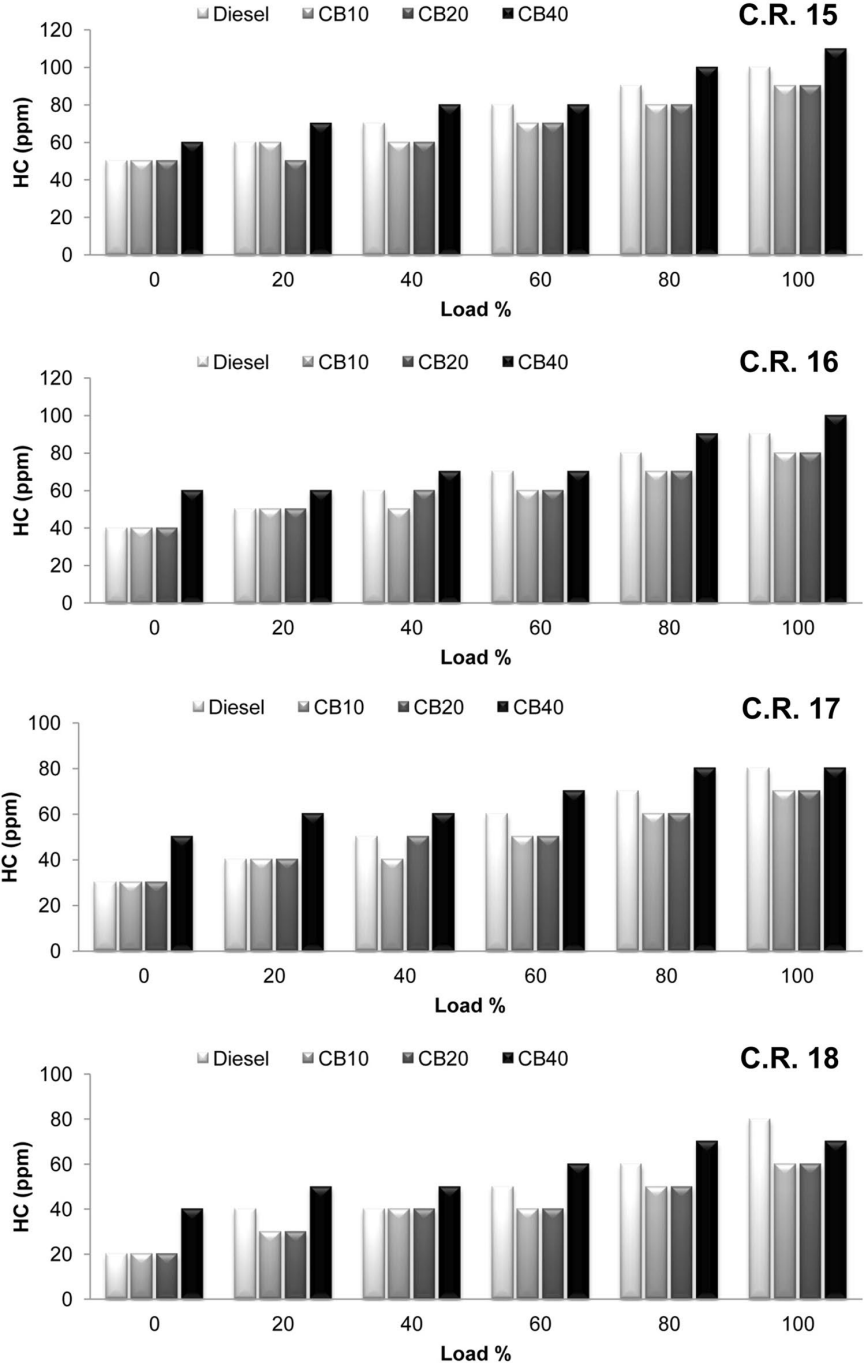
Variation of hydrocarbon with load at compression ratio 15, 16, 17 and 18

**Fig. 6:**
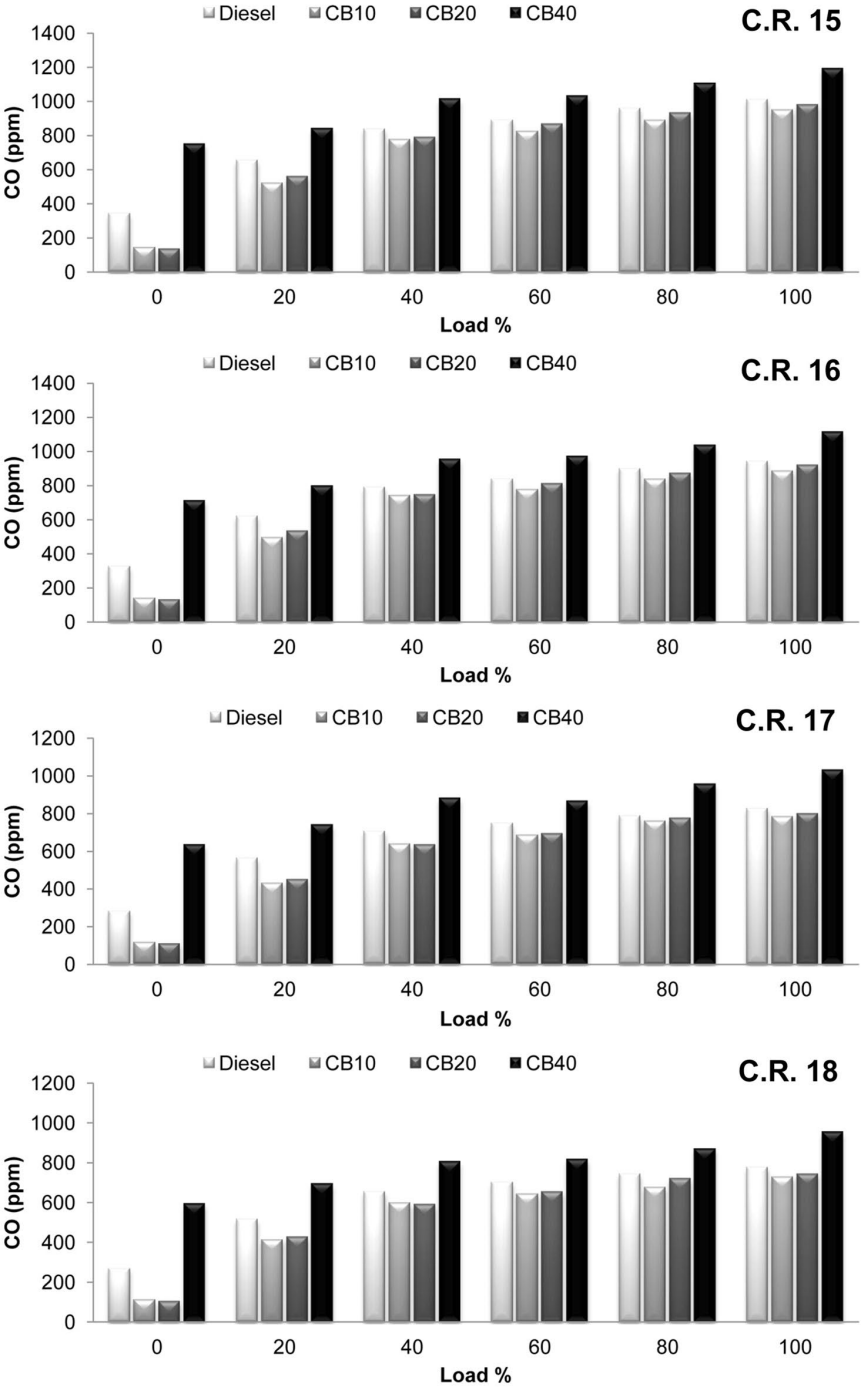
Variation of carbon monoxide with load at compression ratio 15, 16, 17 and 18

Variation of the hydrocarbon (HC) emissions with load at all C.R is shown in Fig. 5. Results show that HC emissions from CB10 and CB20 are comparatively less than diesel fuel by 14 % on an average. In P-θ diagram, attainment of peak cylinder pressure further near to T.D.C in expansion stroke for CB10 and CB20 results in improved combustion process due to less overmixing of fuel and air and contributes to the decrease in HC emissions. HC emissions decreased with increase in compression ratio. On an average HC emissions decreased by 37.84, 42.83, 41.17 and 31.8 % for diesel, CB10, CB20 and CB40 respectively, when C.R is increased from 15 to 18. The result of high temperature due to increase in C.R and the presence of additional oxygen content in biodiesel improves the combustion process resulting in greater decrease in the emissions for biodiesel blends with increase in C.R. For the same reason, similar phenomenon of carbon monoxide (CO) emissions variation with load for biodiesel blends is observed at all C.R and shown in Fig. 6. An average decrease in CO emissions for diesel, CB10, CB20 and CB40 are 21.9, 22.54, 24.46 and 20.22 % respectively when C.R is increased from 15 to 18.

**Fig. 7:**
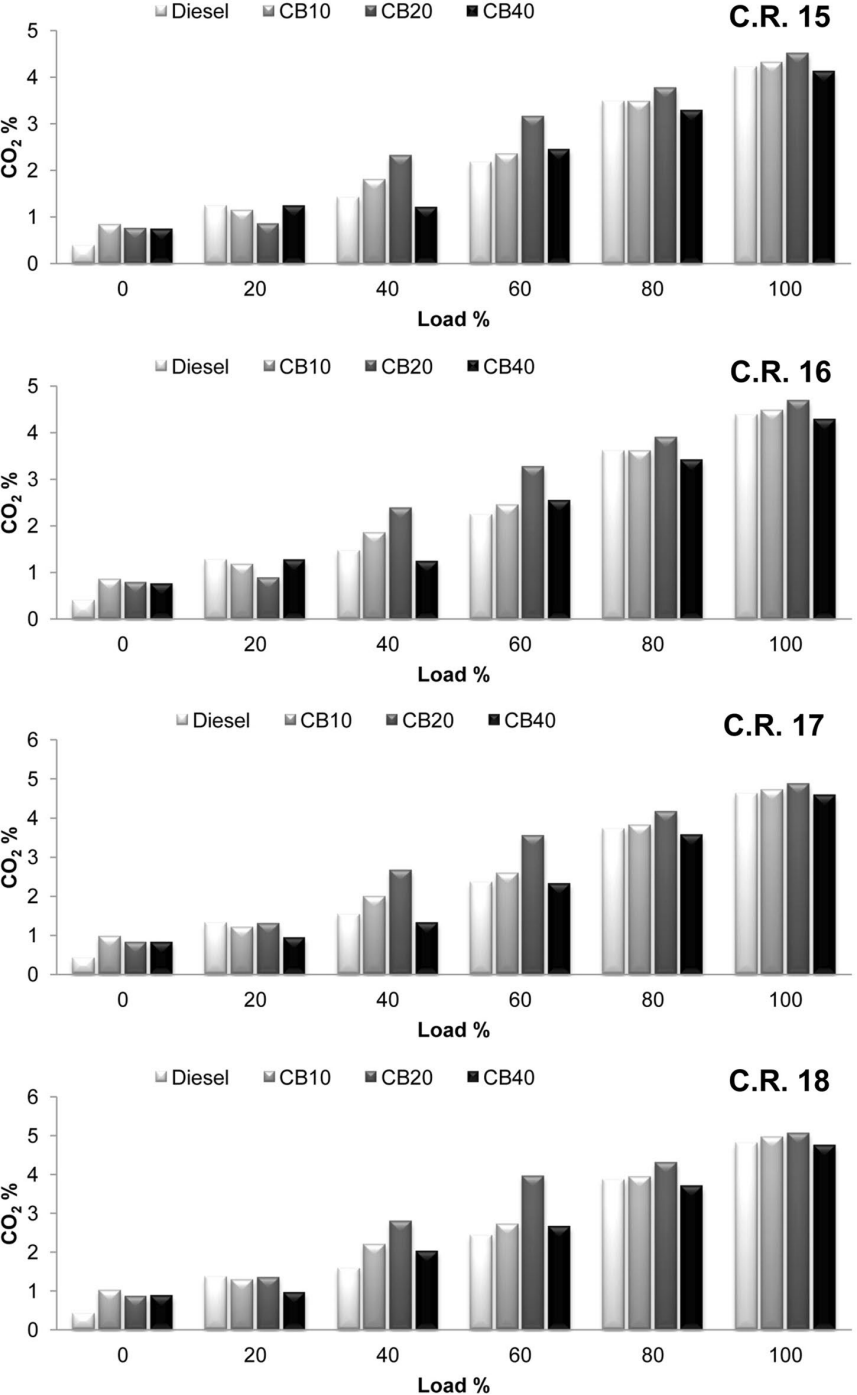
Variation of carbon dioxide with load at compression ratio 15, 16, 17 and 18

**Fig. 8:**
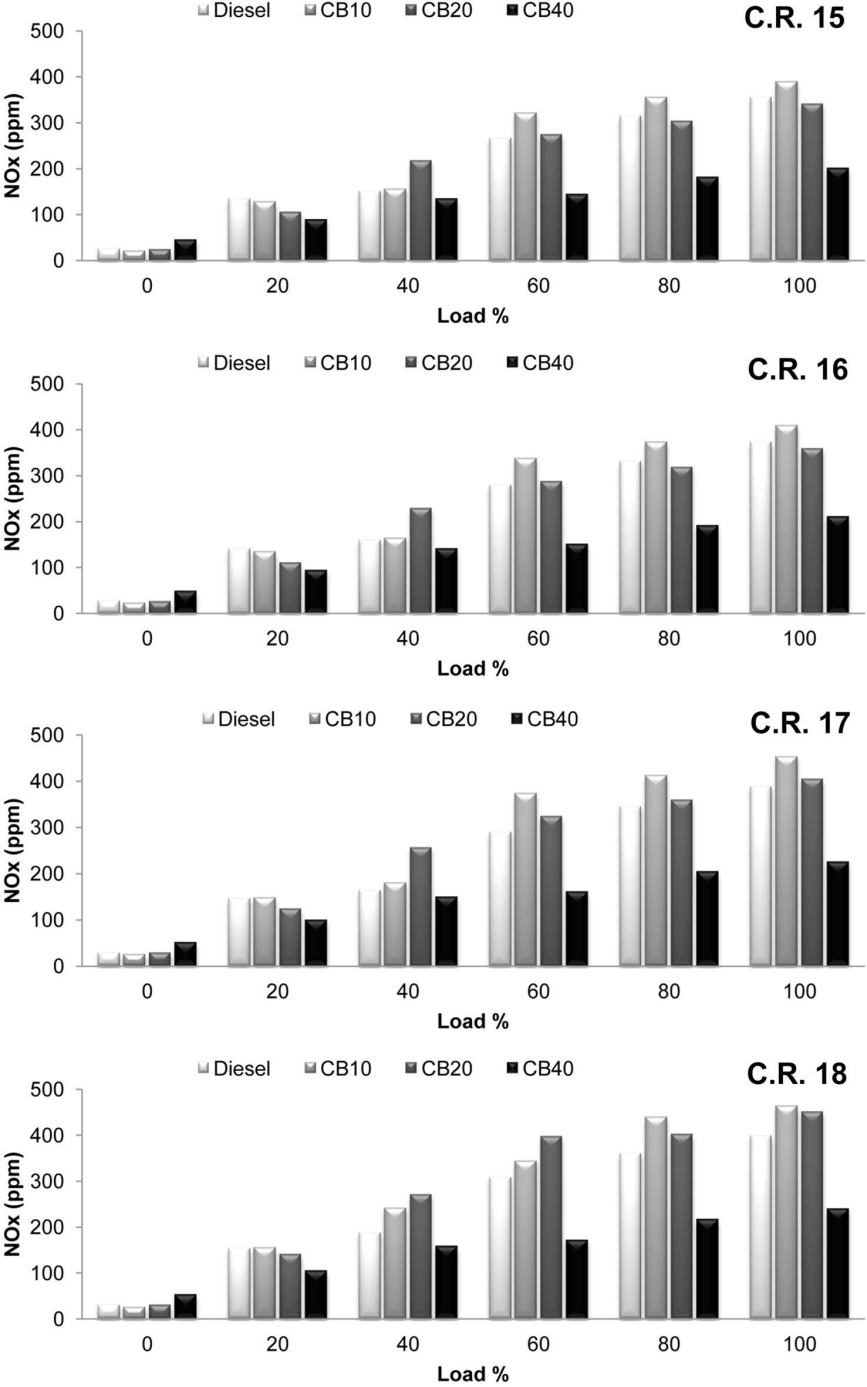
Variation of oxides of nitrogen as NO_x_ with load at compression ratio 15, 16, 17 and 18

As a result of improved combustion due to presence of additional oxygen content in biodiesel, carbon dioxide (CO_2_) emissions increased for crude rice bran biodiesel blends. Variation of CO_2_ emissions with load at all C.R is shown in Fig. 7. Highest CO_2_ emission is observed for CB20 at all C.R. CO_2_ emissions increased with increase in engine load and compression ratio. An average increase of 11.73, 16.9, 24.16 and 16.9 % in CO_2_ emissions for diesel, CB10, CB20 and CB40 respectively, is recorded when C.R is increased from 15 to 18. Improved combustion inside the combustion chamber increases the temperature. Attainment of higher peak pressure near to T.D.C in the expansion stroke for CB10 and CB20 compared to diesel and CB40 results in higher combustion chamber temperature leading to increase in NO_x_ formation The same can be observed in the variation of oxides of nitrogen as NO_x_ with load at all C.R in Fig. 8. Increase in compression ratio increases the combustion temperature which tends to increase NO_x_ formation. NO_x_ emissions increased by an average of 16.11, 24.71, 31.96 and 18.26 % for diesel, CB10, CB20 and CB40 respectively, when C.R is increased from 15 to 18. CB40 has the lowest NO_x_ emission due to its lower cylinder pressure resulting in lower temperature in combustion chamber compared to other fuel blends.

Emissions from oxides of nitrogen (NO_x_) can be controlled by two methods (Teng et al. 2007):

1. Treating the exhaust NO_x_ with a reduction catalyst converter (either selective catalyst reduction or lean NO_x_ trap).
2. Operating the engine with high rate of cooled exhaust gas recirculation (EGR) in order to lower the charge temperature at the start of combustion, as for the full range of engine operations the average NO_x_-reduction efficiency is less than 80 %.

## Conclusion

Conclusions summarized from the above study and experimental investigations are as follows:

1. Blends CB10 and CB20 show almost similar B.S.F.C and B.T.E compared to diesel at all compression ratios. Higher B.S.F.C and lower B.T.E is recorded for higher blends. Increase in B.T.E and decrease in B.S.F.C is observed with increase in compression ratio.
2. Maximum cylinder pressure attained is for CB10 and CB20 than diesel. Cylinder pressure increased with increase in compression ratio.
3. Blends of crude rice bran biodiesel show better emission result than diesel. With the increase in compression ratio from 15 to 18, HC emission decreased by 38.4 %, CO emission decreased by 22.27 % and CO_2_ emission increased by 17.43 % on an average. NO_x_ emissions increased by 22.76 % on an average with increase in compression ratio.

It can be concluded that blends of crude rice bran biodiesel show improved performance and lower emission characteristics than diesel on increasing the compression ratio.

## Abbreviations

FFA: free fatty acid
B.S.F.C: brake specific fuel consumption
B.T.E: brake thermal efficiency
C.R: compression ratio
EGR: exhaust gas recirculation
HC: hydrocarbon
CO: carbon monoxide
CO_2_: carbon dioxide
NO_x_: oxides of nitrogen
T.D.C: Top Dead Centre
A.T.D.C: After Top Dead Centre
WHO: World Health Organisation
AAP: ambient air pollution
VCR: variable compression ratio
DI: direct injection
USB: Universal Serial Bus
